# Preventing Atherosclerotic Cardiovascular Disease in Young Male Androgen Abusers

**DOI:** 10.1002/clc.70257

**Published:** 2026-02-19

**Authors:** Peter Bond, Diederik L. Smit, Tijs Verdegaal, Willem de Ronde

**Affiliations:** ^1^ Department of Performance and Image‐Enhancing Drugs Research Android Health Clinic Utrecht the Netherlands; ^2^ Department of Internal Medicine Zaandam Medical Centre Zaandam the Netherlands; ^3^ Department of Internal Medicine Spaarne Gasthuis Haarlem the Netherlands

**Keywords:** anabolic steroids, androgens, atherosclerosis, testosterone

## Abstract

**Background:**

Androgen abuse among young men is a prevalent yet under recognized risk factor for atherosclerotic cardiovascular disease (ASCVD). Supraphysiological androgen exposure adversely affects lipoprotein profiles, blood pressure, and vascular function, potentially accelerating atherosclerosis even in otherwise healthy individuals. Conventional cardiovascular risk prediction models may underestimate risk in this population due to fluctuating biomarker profiles and other unaccounted risk‐increasing factors that are not captured in these models.

**Objectives:**

To review the mechanisms by which androgen abuse contributes to ASCVD, to highlight limitations of traditional risk stratification tools in this population, and to propose tailored approaches to risk assessment and prevention.

**Methods:**

Narrative review of epidemiological, imaging, and mechanistic studies examining cardiovascular risk factors and subclinical atherosclerosis in androgen abusers.

**Results:**

Available evidence indicates that androgen abuse adversely affects lipid profiles, blood pressure, and vascular endothelial function, and is associated with increased subclinical atherosclerosis. Conventional risk prediction models may underestimate risk in this population due to young age, fluctuating biomarker profiles, and cumulative exposure effects not captured by standard algorithms.

**Conclusions:**

Androgen abuse should be recognized as a cardiovascular risk‐enhancing factor in young men. Individualized risk assessment beyond traditional calculators may be warranted to guide preventive strategies in selected patients.

## Graphical abstract

Androgen abuse is an underrecognized ASCVD risk enhancer in young men. Improved risk assessment—integrating androgen exposure, imaging, and individualized prevention—may help in the management of long‐term cardiovascular risk in this population.

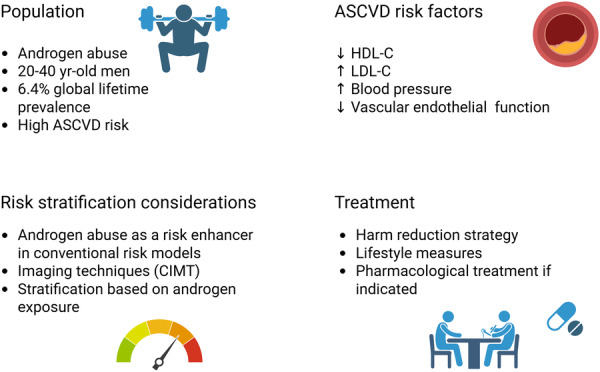

## 1 Introduction

Atherosclerotic cardiovascular disease (ASCVD) remains one of the leading global causes of morbidity and mortality. Since the seminal findings of the Framingham Heart Study in the 1950s [1], decades of concerted scientific effort have significantly expanded our knowledge of the underlying pathophysiology. We can now identify and stratify those at risk by screening for a family history of premature ASCVD, applying biomarker‐based risk algorithms (e.g., SCORE2), and utilizing imaging modalities such as coronary artery calcium (CAC) scoring. Subsequently, we can reduce ASCVD risk by lifestyle modifications and the use of a range of safe and effective pharmacological interventions. Various preventive guidelines have been published to support the clinician in the collective battle against ASCVD [2, 3, 4, 5].

While ASCVD events are most common in the elderly, young adults are not exempt from ASCVD as certain risk factors, in particular genetic predisposition, can markedly accelerate atherosclerotic development. This is especially evident in individuals with homozygous familial hypercholesterolemia, characterized by very high LDL cholesterol (LDL‐C) levels since birth, in whom cardiovascular disease may manifest in childhood [6]. Besides genetic predisposition, certain lifestyle choices can also increase the risk of ASCVD, such as tobacco smoking, a sedentary lifestyle, a diet high in saturated fats and low in fiber, whether or not combined with a high caloric intake leading to obesity, and high alcohol consumption. Recreational drug use may also expedite ASCVD (e.g., amphetamines) [7]. With an estimated global lifetime prevalence rate of 3.3% (6.4% for males and 1.6% for females) [8], androgen abuse, defined as the illicit use of androgens in supratherapeutic dosages, may represent another widely prevalent and important risk factor for ASCVD [9, 10]. While androgen abuse was initially confined to professional athletes for performance enhancement, a shift in user demography occurred in the late 1970s and early 1980s toward increased recreational use in the eye of image enhancement [11]. Typical abusers are 20‐ to 40‐year‐old male bodybuilders, fitness enthusiasts, or martial arts athletes pursuing increases in muscle mass or strength [12]. Indeed, androgens are highly effective for this purpose [13] and the rarity of disastrous negative effects on the short‐term is unlikely to deter from their abuse [14]. Long‐term effects, however, might include accelerated atherosclerotic development [10, 15].

Androgen abuse often entails high‐dose cycles of several months followed by periods of no use that can span several weeks to months (intermittent cycling) or alternating high‐dose cycles with a lower maintenance dosage for extended periods (“blast‐and‐cruise”) [16]. Weekly doses can range from 250 mg to several grams and frequently involve the concurrent use of multiple androgens—a practice known as “stacking” [12]. Injectable compounds are used by most and are often combined with oral 17α‐alkylated androgens for varying durations. This pattern of use may persist for years, sometimes decades, and carries substantial health risks [9].

In this article, we will discuss the relation between androgen abuse and ASCVD and provide considerations for tailored risk stratification in this ASCVD‐prone population and treatment options.

### 1.1 Androgen Abuse and ASCVD Risk

Unlike many other countries, where doping tests are restricted to competitive athletes, introducing selection bias by excluding recreational users, Denmark and Sweden conduct doping tests in commercial gyms. This broader testing approach facilitates more generalizable conclusions about doping effects on various health outcomes by using data from national health registries. Swedish and Danish retrospective cohort studies show increased cardiovascular morbidity and mortality in individuals who tested positive for androgen abuse [17, 18]. In the Danish cohort, percutaneous coronary intervention (PCI) or coronary artery bypass graft (CABG) and acute myocardial infarction were three times more prevalent in androgen abusers than in matched controls [18]. The Swedish cohort reported an insufficient number of cardiovascular events for statistical analysis, although the numbers also suggest increased ASCVD burden [17]. Importantly, these studies have a comparatively short follow‐up time (mean 4−11 years) with a positive doping test at a relatively young age (mean < 30 years). As atherosclerosis can take decades to clinically manifest, these studies likely underestimate the lifetime ASCVD burden caused by androgen abuse.

Before manifesting clinically as a myocardial infarction or ischemic stroke, atherosclerosis can be detected by imaging techniques such as computed tomography (CT) or ultrasound. A coronary calcium scan quantifies calcification of the coronary arteries by reporting an Agatston score. However, the vast majority of people under the age of 45 years will have an Agatston score of 0 [19]. This is not surprising as calcification only occurs in advanced lesions and thus calcified plaques are less common in the relatively young. Indeed, an Agatston score > 0 was detected in only 18% of an asymptomatic cohort with a mean age of 46 years, while atherosclerotic plaque was evident by ultrasound in 63% [20]. With the use of contrast media, however, noncalcified intraluminal plaque can be detected on a coronary CT angiogram (CCTA). This allows for a more sensitive assessment of less advanced subclinical atherosclerosis. In a small‐scale cross‐sectional study using this technique, 25% of young men using androgens (29 years ± 5 years) had plaques in at least two coronary arteries, while none were found in any of the strength‐trained and sedentary control groups [21]. A larger cross‐sectional study showed higher plaque volumes in androgen abusers compared with nonusers, and both plaque volume and coronary calcium score demonstrated a significant positive correlation with lifetime androgen abuse [15]. Notably, therapeutic testosterone treatment increased noncalcified coronary artery plaque volume in older men compared with placebo [22], raising the possibility that similar effects may occur in younger men, although this has yet to be established. However, especially in young adults, CCTA may not fully reflect atherosclerotic burden. Early atherosclerosis is not visible due to arterial wall expansion prior to plaque formation encroaching on the lumen (also known as the Glagov phenomenon) [23]. Consequently, extraluminal plaques remain out of sight. Furthermore, small plaque volumes can be difficult to identify on CCTA, although efforts are being made to substantially improve detection by machine learning [24].

Early atherosclerosis, characterized by arterial wall expansion, that is, thickening of the intima‐media layer, can be visualized using noninvasive B‐mode ultrasound [25]. The distance between the intimal‐luminal and medial‐adventitial interfaces, representing the intima‐media thickness (IMT), can be accurately measured. This is most commonly done in the carotid artery (CIMT) due to its easily accessible location and standardized measurement protocols. A small cross‐sectional study with 10 androgen‐using and 10 non‐using male bodybuilders showed no difference in CIMT between groups [26]. A recent larger cross‐sectional study with 56 users and 67 nonusers did show higher CIMT (+0.05 mm) in the former group [27]. The differences in results between these two studies might be due to small sample size of the first. Furthermore, the subjects in the recent study had used androgens on average for 11 years—a relatively long duration of exposure—whereas this was unreported in the other trial. If the total time of androgen exposure in the other study was short, it might have been insufficient to result in detectable differences in CIMT.

Androgen abuse negatively impacts serum lipoproteins, and this likely contributes significantly to these worrying observations. In controlled trials, supraphysiological dosages of testosterone enanthate (up to 600 mg weekly) decrease HDL cholesterol (HDL‐C) in some [28, 29, 30, 31], but not all trials [13, 32, 33]. HDL‐C remained unchanged in controlled trials administering nandrolone decanoate (100–200 mg weekly) [34, 35, 36]. The null findings on HDL‐C in these trials likely have to do with the relatively low dosages and small sample sizes resulting in type II errors (false negatives) [9]. The HAARLEM study, an observational cohort study of self‐administered androgen cycles in 100 men, found a 0.4 mmol/L (33%) decrease in HDL‐C during use [37]. A review of predominantly small prospective cohorts of self‐administering androgen abusers also revealed an average HDL‐C decrease of 52% [38]. Notably, orally bioavailable androgens in particular are consistently associated with strong suppression of HDL‐C [30, 33, 39, 40, 41, 42, 43]. The HDL‐suppressive effect can last for over a month after last administration [44], and HDL‐mediated cholesterol efflux capacity also decreases [21]. The underlying mechanism for decreased HDL‐C levels is unclear, but it is generally thought to involve increased hepatic lipase (HL) activity [30, 31, 32, 33]. A case report of two HL‐deficient brothers, however, suggests that this might only partially explain the decrease, since stanozolol administration decreased their HDL‐C levels by 20% regardless [45]. LDL‐C levels are generally unaffected by injectable androgens such as testosterone enanthate (up to 600 mg weekly) and nandrolone decanoate (up to 200 mg weekly) [13, 28, 29, 31, 32, 33, 35, 36], although this may also be an artifact of the androgen dosages used that may not represent real‐world usage. Again, orally bioavailable androgens consistently show increases in LDL‐C [30, 33, 39, 40, 43]. The HAARLEM study showed a 0.4 mmol/L (14%) increase in LDL‐C relative to baseline during androgen abuse. The modest average increase likely reflects the relatively low use of oral androgens in this cohort (30% of participants), and oral use indeed correlated with a greater increase in LDL‐C [37]. The previously cited review reports an average LDL‐C increase of 36% [39]. Little data exists on apolipoprotein B, although the effect of androgens on its serum concentration similarly shows a dichotomy between injectable and orally bioavailable androgens, with use of the latter leading to an increase [30, 37].

Evidence from in vitro, animal, and human studies shows an increased expression of 3‐hydroxy‐3‐methylglutaryl coenzyme A (HMG‐CoA) reductase [46, 47, 48]—the rate‐limiting enzyme in cholesterol synthesis through the mevalonate pathway and the therapeutic target of statins. Additionally, HL acts on intermediate‐density lipoprotein (IDL) particles, generating the smaller LDL‐C particles, as well as decreasing the size of the existing larger LDL I and II particles into the smaller LDL III and IV subclasses [49], which are considered more atherogenic due to their greater arterial wall penetration and longer plasma residence time. The net effect might be a disproportionate increase in LDL particles relative to LDL‐C, leading to discordance between LDL particle number and LDL‐C.

Interestingly, androgens may decrease lipoprotein (a) (Lp(a)) [9]. Lp(a) is an LDL‐like particle in which the apolipoprotein B moiety is covalently linked to apolipoprotein (a) by a disulfide bond. The polymorphism of apolipoprotein (a), in particular the variation in the kringle IV type 2 repeat domain, is an important determinant of Lp(a) concentrations. Consequently, the number of kringle IV type 2 repeats, which varies from 2 to more than 40, positively correlates with Lp(a) plasma concentrations [50]. Lp(a) concentrations are for over 90% genetically determined [51] and approximately one in five has elevated concentrations associated with increased CVD risk [50]. Although not widely adopted in primary prevention guidelines, the European Atherosclerosis Society recommends testing Lp(a) concentration at least once in adults in a consensus statement [52]. While androgens decrease Lp(a), this is irrelevant for the majority of the population that does not have elevated Lp(a) levels, and additionally, it is unclear to what extent a Lp(a) decrease affects health outcomes. Importantly, an Lp(a) measurement during androgen abuse is therefore not representative of someone's true Lp(a) levels, which might erroneously lead to the conclusion that someone is not at elevated CVD risk, as this value might be elevated when a user is “off.”

Increased blood pressure might further contribute to accelerated atherosclerosis, although the underlying mechanism remains unclear [53]. The HAARLEM study showed an increase of 7 and 3 mmHg in systolic and diastolic blood pressure, respectively, during androgen abuse [37]. While this increase may appear modest, it is comparable in magnitude—albeit in opposite direction—to that of a single blood pressure‐lowering medication, such as an ACE inhibitor or angiotensin receptor blocker, and thus its detrimental effect should not be underestimated. Importantly, while some androgen abusers will experience minimal or no rise in blood pressure, others will experience more pronounced elevations. Various mechanisms have been proposed to explain the increase in blood pressure by androgens. This includes vasoconstriction via upregulation of thromboxane A2 expression, norepinephrine synthesis, angiotensin II expression, and endothelin‐1 action [54]. Some studies also suggest that production of 20‐hydroxyeicosatetraenoic acid (20‐HETE; an eicosanoid and arachidonic acid metabolite generated by CYP4A/4 F) in vascular smooth muscle cells drives androgen‐dependent hypertension [55]. Since both androgen and estrogen receptors are expressed throughout the vasculature [56], it is possible that non‐aromatizing androgens differ from aromatizing androgens, such as testosterone, in their effects on blood pressure.

Several studies have investigated vascular endothelial function by measuring flow‐mediated dilation (FMD) [26, 57, 58, 59, 60] and carotid artery reactivity (CAR%) [60] in androgen abusers. FMD is based on the premise that a conduit artery dilates in response to increased shear stress from elevated blood flow through it, a process that is attenuated in the presence of endothelial dysfunction. With the exception of one study [26], FMD in AAS users is decreased compared with controls [57, 58, 59, 60]. CAR% similarly measures dilation of a conduit artery, albeit in response to sympathetic activation rather than shear stress. CAR% was also decreased in AAS users compared with controls [60]. Nevertheless, all studies to date have employed a cross‐sectional design, which limits causal inference and warrants further investigation through interventional designs.

## 2 Considerations for Risk Stratification

### 2.1 Limitations of Conventional Risk Models

Most androgen abusers are young males (20−40 years of age) [12] and are thus, virtually by definition, at low 10‐year CVD risk. Additionally, risk algorithms, such as SCORE2 and PCE, are validated for those 40 years of age and older. However, this does not absolve us from clinical reasoning as to why this population might qualify for primordial prevention, that is, early treatment of modifiable risk factors.

Although lifestyle modifications are recommended for young adults, pharmacological intervention is generally not recommended in primary prevention guidelines. Given that atherosclerosis begins early in life and that safe, effective, and inexpensive drugs are available, and screening is similarly easily accessible and inexpensive, several experts in the field of preventive cardiology have called for earlier and more intense treatment of ASCVD [61, 62]. As Peter Libby noted in his recent review on atherosclerosis in Nature [63]: “If the entire population maintained LDL‐C concentrations akin to those of a neonate (or to those of adults of most other animal species), atherosclerosis might well be an orphan disease.” This line of progressive thinking holds especially true for populations with an increased risk at a relatively young age, such as androgen abusers. This shift in preventive strategy, notwithstanding, while supported by emerging evidence, has the potential of overmedicalization in low‐risk individuals.

Mounting evidence supports the LDL cumulative exposure hypothesis which proposes that [64]: “the size of the accumulated plaque burden, the rate of plaque progression and the corresponding absolute risk of having an acute cardiovascular event at any point in time for an individual are determined by their cumulative exposure to LDL.” This implies that ASCVD risk can be estimated by calculation of cumulated LDL exposure, expressed in “plaque–years” (measured in mmol/L or mg/dL), and that risk reduction is most effectively achieved by reducing LDL‐C levels as low and as early as possible.

LDL‐C levels are lowest during late adolescence and then increase roughly by 50% over time before leveling off by age 45 in men and 60 years in women [65]. A single LDL‐C measurement, therefore, provides only a rough estimate of cumulative lifetime exposure. Importantly, in long‐term androgen abusers, LDL‐C levels may fluctuate significantly over time due to the androgen‐induced increases superimposed on age‐related trends. An LDL‐C measurement taken during an androgen abuser's “off” period may underestimate cumulative exposure and, consequently, lifetime ASCVD risk. Another important consideration is that the same LDL‐C exposure (level × time) confers greater ASCVD risk when it occurs early in life [66, 67, 68]—a particularly relevant observation as androgen abuse is typically initiated in early adulthood. Moreover, the extent to which the strong HDL‐C suppression affects atherosclerosis progression remains unclear. Indeed, if we assume a 33% LDL‐C increase from a baseline level of 3 mmol/L, this results in an absolute increase of 1 mmol/L, adding 10 plaque–years over a decade. While significant, it does not fully explain the degree of ASCVD burden observed in cohort studies.

### 2.2 A Case for Imaging‐Based Risk Assessment

These findings suggest that traditional serum biomarker‐based risk estimation in androgen abusers may be inaccurate, particularly in former androgen abusers or in active users during an “off” period. Imaging techniques, such as CIMT measurement, may provide a valuable adjunct to risk stratification. Current guidelines do not recommend CIMT measurement for risk prediction in the general population, primarily because it adds little prognostic value over current algorithms [69]. However, CIMT measurement might be more useful in this population, as risk prediction algorithms are based on serum biomarkers that are less reliable in androgen abusers. As a noninvasive, inexpensive low‐cost technique, the CIMT value ≥ 75th percentile of age‐ and sex‐specific reference values serves as an appropriate cut‐off to consider an individual high‐risk [70]. While a single CIMT measurement may offer limited prognostic value, longitudinal tracking—such as annual measurements—may enhance its utility by capturing atherosclerosis progression over time. Although this approach lacks formal validation, it could support clinical decision‐making in selected cases. Furthermore, the CIMT may also serve as a communication tool to illustrate subclinical atherosclerosis to androgen abusers and support counseling aimed at cessation, although this remains hypothetical and is not yet supported by empirical data.

### 2.3 Incorporating Androgen Use Into Existing Prediction Models

Another option would be to include androgen abuse as a risk modifier within existing models based on the population prevalence and the subdistribution hazard ratio (SHR) of androgen abuse. While this approach simplifies a highly heterogeneous exposure group, as there is a wide variability in dose, duration, and compounds used, it may still offer practical value in the absence of more individualized risk models; an approximate adjustment is arguably preferable to none. A lifetime risk model, such as LIFE‐CVD2 [71], may be especially suitable and has the added benefit of supporting an age range of 35–100 years. This approach has recently been proposed to increase the applicability of prediction models for those with known additional risk modifiers [72]. The following formula might then be used to recalculate risk for androgen abuse:

Adjusted risk = 1 – (1 – individual predicted risk)^(SHR/population relative risk)^

Unfortunately, no SHRs are published for CVD risk due to androgen abuse in the literature. However, working under the assumption of rare competing and non‐differential risks, the HR might serve as a reasonable replacement. We believe that this is justified in young androgen abusers since CVD events and mortality are very low in this age group, and thus, the SHR is probably not much different from the HR. The earlier‐mentioned Danish cohort did not report cardiovascular morbidity as a composite endpoint, but individually reported adjusted HRs of cardiovascular events ranged from 2.26 to 8.90 [18]. A conservative estimate of 2.0 could therefore be used as an SHR for risk adjustment—acknowledging that this likely underestimates risk. In turn, the population relative risk (RR) can be calculated using the SHR and prevalence. A comprehensive meta‐analysis reports a global lifetime prevalence rate of androgen abuse for males of 6.4% [8], yielding a population RR of 1.064. If we take an individual predicted 10‐year risk of 5% this would increase to an adjusted risk of 9.2% if the patient is a male androgen abuser.

### 2.4 Androgen Exposure as a Continuous Risk Metric

An alternative approach would be to conceptualize androgen abuse as a continuous risk variable, based on cumulative exposure above a supraphysiological threshold, for example, ≥ 250 mg of androgens per week. This model would resemble the concept of pack–years in smoking‐related risk estimation, where both intensity and duration are factored into long‐term exposure. Users could be stratified into exposure categories such as mild (< 2 years), moderate (2–5 years), moderately heavy (5–10 years), and heavy (> 10 years) cumulative use. While this method may allow for more individualized and equitable risk assessment and therapeutic decision‐making, it presents several limitations. First, the precise relationship between cumulative AAS exposure and ASCVD risk remains poorly quantified, both in epidemiological studies and in existing risk models. Second, accurate assessment requires a detailed drug history, typically obtained through a nuanced interview by a clinician familiar with androgen abuse patterns, compounds, and dosing schemes. Despite these challenges, framing androgen abuse in terms of cumulative burden may better reflect the heterogeneity of risk in this population than binary classification alone.

### 2.5 Treatment

Most health benefits might be obtained by convincing the androgen abuser to discontinue the abuse. Androgen abusers should therefore be extensively counseled about the health risks involved and continued use should be strongly discouraged. This needs to be addressed in an unbiased and open manner in order to establish trust with the patient, as fear of judgment might deter the androgen abuser from seeking healthcare in the future. A framework for engaging with androgen abusers in clinical practice as provided by Smit et al. could serve as a helpful starting point to aid the clinician [73]. If cessation of abuse is unattainable, a harm reduction strategy approach as outlined by Smit et al. can be applied [16]. This harm reduction approach focuses on reducing cycle duration, cycle dosage, the number of compounds (with emphasis on avoiding oral androgens due to their greater hepatotoxicity and disturbance of lipid profiles), and extending recovery time between cycles. Additionally, implementing lifestyle measures to improve risk factors should be encouraged. While physical activity is usually adequate in this population, improvements may still be made by advising other general lifestyle measures as outlined in clinical guidelines, such as avoiding trans fat, limiting added sugar, saturated fat, and alcohol intake, increasing dietary fiber intake, and smoking cessation.

Cardiovascular risk management may be appropriate for patients who are unwilling to discontinue their androgen abuse. Given the limitations of conventional risk prediction tools in androgen abusers, treatment decisions should not rely on traditional algorithms alone. Instead, one of the alternative approaches outlined above may help inform individualized clinical judgment. In particular, cumulative androgen exposure, expressed as years of supraphysiological use in conjunction with imaging data, may provide a more accurate reflection of cardiovascular risk in this population. Preventive pharmacotherapy may be appropriate for individuals with a moderate‐to‐heavy androgen history (i.e., > 5 years at ≥ 250 mg/week), relatively short but ongoing abuse without intent to discontinue, or a CIMT above the 75th percentile or with progressive subclinical atherosclerosis on serial measurements. Additional risk‐enhancing features, such as a positive family history of premature ASCVD, should further guide decision‐making. This approach enables better risk differentiation and more targeted use of lipid‐lowering or blood pressure‐lowering therapies in otherwise young and asymptomatic individuals. For example, while the 2021 ESC guidelines on primary prevention recommend an LDL‐C target of < 2.6 mmol/L for apparently healthy individuals under 50 years of age [2], the presence of moderate‐to‐heavy androgen exposure may warrant reclassification into a higher cardiovascular risk category, justifying a more stringent target of < 1.8 mmol/L instead.

When preventative pharmacological treatment is indicated, statin therapy remains the cornerstone of first‐line pharmacological dyslipidemia treatment. However, a substantial number of patients are hesitant to initiate statin therapy. In our experience, a primary concern among this population is the occurrence of muscle‐related complaints associated with statin use. In randomized‐controlled trials in older individuals, these complaints are uncommon [74], yet there is a relatively high incidence in observational studies and in practice—likely due in large part to the nocebo effect [75]. Nevertheless, these complaints may be more prevalent in those regularly engaging in physical exercise [76]. For this purpose, it might be preferable to start androgen abusers on a low‐dose hydrophilic statin (e.g., rosuvastatin 5 mg daily), given their greater hepatoselectivity and consequently purported lower propensity for muscle‐related complaints. If symptoms occur, it is prudent to attempt switching to a different statin (e.g., simvastatin), reduce the dose, or adopting an alternate‐day regimen [77]. If side effects remain, alternative therapies such as ezetimibe, bempedoic acid, and proprotein convertase subtilisin/kexin type 9 (PCSK9) inhibitors should be explored. Given the potential discordance between LDL‐C concentration and particle number in androgen abusers, targets based on apolipoprotein B or non‐HDL‐C may offer a more reliable risk reduction strategy in selected individuals.

To treat hypertension, an angiotensin‐converting enzyme (ACE) inhibitor or an angiotensin II receptor blocker (ARB) is preferred because they do not affect exercise capacity [78]. Alternatively or additionally, calcium channel blockers or thiazide‐like diuretics are preferred. Given that the increase in blood pressure by androgen abuse is relatively modest [37], secondary causes of hypertension other than androgen abuse should still be sought in those younger than 30 years or when hypertension persists during “off” periods or when androgen abuse is limited to a testosterone maintenance dose. A proper blood pressure cuff size is essential when measuring blood pressure, as androgen abusers are more likely to have large upper arm circumferences. Using an inappropriately small cuff may result in overestimated readings [9].

It is intuitively reasonable to assume that the aforementioned pharmacological treatments for dyslipidemia and hypertension are similarly effective in androgen abuse as in the general population. However, empirical data specific to this population are currently lacking. Given their elevated cardiovascular disease risk, future research exploring the efficacy of these interventions in the context of androgen abuse would therefore be particularly valuable.

## 3 Conclusion

Available cohort studies and mechanistic data indicate that androgen abusers—particularly young men using supraphysiological doses—carry a significantly elevated risk of ASCVD. While dyslipidemia and hypertension are two evident, modifiable risk factors, additional pathways such as endothelial dysfunction may contribute to disease development. In this population, traditional risk calculators often fail to reflect the true burden due to age‐based underestimation and fluctuating biomarker profiles. A more nuanced approach to prevention is therefore warranted, integrating cumulative androgen exposure, imaging findings, and family history. Such an individualized strategy may help to identify those who would benefit most from early pharmacologic and lifestyle interventions. In this context, primordial prevention is not only appropriate but also arguably necessary to mitigate long‐term cardiovascular harm. While trials supporting this approach are obviously lacking, it should be appreciated that therapeutic inertia is likely more harmful than potential overtreatment of ASCVD risk factors in this at‐risk population.

## Author Contributions

**Peter Bond:** conceptualization and writing – original draft. **Diederik L. Smit:** writing – review and editing. **Tijs Verdegaal:** writing – review and editing. **Willem de Ronde:** writing – review and editing.

## Conflicts of Interest

The authors declare no conflicts of interest.

## Data Availability

Data sharing is not applicable to this article as no data sets were generated or analyzed during the current study.
